# Association between Behavioral Ambidexterity and Brain Health

**DOI:** 10.3390/brainsci10030137

**Published:** 2020-02-29

**Authors:** Keisuke Kokubun, Yoshinori Yamakawa, Kazuo Hiraki

**Affiliations:** 1 Open Innovation Institute, Kyoto University, Kyoto 606-8501, Japan; yamakawa@bi-lab.org; 2 ImPACT Program of Council for Science, Technology and Innovation (Cabinet Office, Government of Japan), Chiyoda, Tokyo 100-8914, Japan; 3 Institute of Innovative Research, Tokyo Institute of Technology, Meguro, Tokyo 152-8550, Japan; 4 Office for Academic and Industrial Innovation, Kobe University, Kobe 657-8501, Japan; 5 NTT Data Institute of Management Consulting, Inc., Chiyoda, Tokyo 102-0093, Japan; 6 Graduate School of Arts and Science, The University of Tokyo, Tokyo 153-8902, Japan; khiraki@idea.c.u-tokyo.ac.jp

**Keywords:** gray-matter brain healthcare quotient, neuroimaging data, MRI, ambidexterity, exploration, exploitation, curiosity, grit, self-efficacy, gray matter

## Abstract

Appropriately handling and switching exploration of novel knowledge and exploitation of existing knowledge is a fundamental element of genuine innovation in society. Moreover, a mounting number of studies have suggested that such “ambidexterity” is associated not only with organizational performance but also with the human brain. Among these reports, however, there have not been any definitive MRI-based parameters that objectively and easily evaluate such ambidexterity. Therefore, an MRI-based index derived from gray matter volume, called the gray-matter brain healthcare quotient (GM-BHQ), was used to measure the association between ambidexterity and the entire human brain. For this purpose, 200 healthy adults were recruited as subjects to undergo structural T1-weighted imaging and to answer multiple psychological questionnaires. Ambidexterity was evaluated using two scales: the Curiosity and Exploration Inventory II and the Short Grit Scale, as exploration–exploitation indicators of curiosity and grit, respectively. Additionally, to enrich the understanding of these associations, three additional positive thinking scales were used—the General Self-Efficacy Scale, the Rosenberg Self-Esteem Scale, and the Life Orientation Test—to evaluate self-efficacy, self-esteem, and optimism, respectively. The authors discovered the GM-BHQ was weakly associated with curiosity, grit, and self-efficacy individually after controlling for age and sex. Furthermore, the GM-BHQ was directly associated with curiosity but indirectly associated with grit in the path model. However, no significant association was found between the GM-BHQ and the other outcome indicators (i.e., self-esteem and optimism). These results suggest that brain health is weakly associated with ambidexterity evaluated using psychological tests.

## 1. Introduction

Organizational ambidexterity, defined as the capacity of a business entity to efficiently handle its current business responsibilities and at the same time be flexible to the dynamic environment, has gained attraction as of late [1,2], especially from those working in time-sensitive environments [3]. Recently, researchers have begun to argue that ambidexterity is not only essential at the organizational level but also at the individual level because individuals need to engage in both explorative and exploitative behaviors if they want to be truly innovative in society [4,5]. For example, it is predominantly believed that important decision makers should be able to handle and switch exploratory and exploitative efforts appropriately [6], because responding to dynamic environments requires business leaders to seek emerging knowledge domains while utilizing existing knowledge and core competencies [1].

A mounting number of experimental research investigations have shown that the brain is linked with ambidexterity [7,8,9,10]. However, these findings based on regional brain conditions look complicated and difficult for a person unfamiliar with brain sciences, thus reducing research transferability. In this sense, using a different approach, it was shown that a neuroimaging-derived measure, the gray-matter brain healthcare quotient (GM-BHQ), which is an average of standardized gray matter measures for 116 brain regions based on the Automated Anatomical Labeling atlas [11], could be related not only to age [12] but also to stress and fatigue [13]. Up until now, an MRI-based measure that is able to assess ambidexterity with similar convenience has seemed to be nonexistent. In this study, therefore, we examined the relationship between the GM-BHQ and ambidexterity in healthy participants, hypothesizing that the GM-BHQ could vary due to exploration and exploitation.

In the present study, we used two psychological scales―the Curiosity and Exploration Inventory II [14] and the Short Grit Scale [15]―to measure curiosity and grit, respectively, to evaluate participants’ ambidexterity. Here, curiosity―the desire to learn about what is unknown [14]―and grit―the ability to persevere with a task for a long period of time until it is mastered [15]―refer to proximities of exploitation and exploration, respectively. In fact, previous research supports this approximation, indicating a linkage between curiosity and exploration behavior [16], and grit with exploration and exploitation behaviors [17]. This is because curiosity is related to the intrinsic motivation to learn [18] and has been found to be a significant predictor of occupational/workplace behavior such as job performance [19] and worker innovation [20]. In the field of neuroscience, highly inquisitive primates exhibited thicker gray matter in the precuneus area of the brain, which is known to play a primary part in highly complex human tasks such as periodic and symbolic memory and introspection, than less curious monkeys [21]. Similarly, grit is linked to successful completion of courses in jobs and schooling [22] and, therefore, to success in careers [22,23] and education [24,25]. In the field of neuroscience, Wang et al. [26] found that higher levels of grit are associated with higher regional gray matter volume (GMV) in the right putamen, which is an area known to be involved in reward-based motivation and learning [27,28].

Additionally, to enrich our understanding of these associations, three additional psychological scales were used—the General Self-Efficacy Scale (GSE) [29], the Rosenberg Self-Esteem Scale [30], and the revised version of the Life Orientation Test [31]—for measuring self-efficacy, self-esteem, and optimism, respectively. These scales are valuable because they are designed to evaluate positive *thinking* but do not include the meanings of exploration or exploitation, which in this sense makes a good contrast with two other variables of ambidexterity. Self-efficacy, the confidence in one’s ability to achieve a desired outcome [32], has been found to be associated with better performance in studies [33], sport [34], work [35], and health-promoting behaviors related to diet and exercise [36]. Self-esteem―an individual’s subjective view of his or her own value, which involves a variety of beliefs about the self―is associated with satisfaction with one’s life and job, fewer interpersonal problems, and fewer psychological problems such as anxiety and depression [37]. Optimism, the expectation of positive outcomes, has been prospectively associated with improved well-being and academic progress [38], lower job stress [39], and lower levels of depression [40].

**Figure 1:**
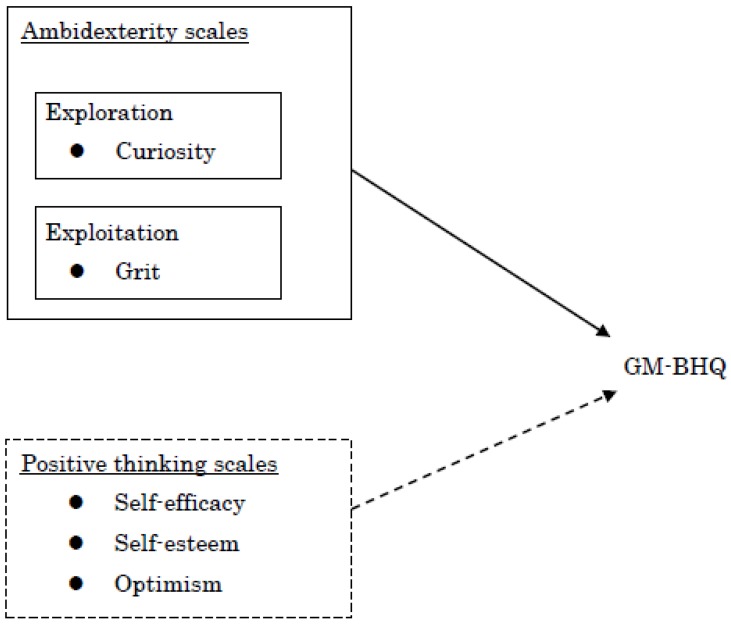
Expected association between psychological scales and the gray-matter brain healthcare quotient (GM-BHQ).

The primary scope of the individual psychological scales is described in Figure 1. Ambidexterity scales were separated into two categories: exploration (curiosity) and exploitation (grit). Positive thinking scales were designed to measure self-efficacy, self-esteem, and optimism. Considering the variable characteristics, we predicted that positive thinking scales would have no association with GM-BHQ in contrast with ambidexterity scales.

## 2. Materials and methods

### 2.1. Subjects

A total of 209 healthy participants (101 females, 108 males) were recruited in Kyoto, Tokyo, and Kobe, Japan. Prospective subjects with any record of neurological, psychiatric, or other medical conditions that may impact the central nervous system were not recruited. Nine participants were excluded after the initial screening because they inadequately answered the questionnaire or did not fulfill requirements for MRI experiments. Thus, the analysis included 200 participants (97 females, 103 males), 20–68 years of age (mean ± standard deviation (SD) age, 44.4 ± 12.2 years). This study was approved by the Ethics Committees of Kyoto University (Kyoto, Japan; approval number 27-P-13), the University of Tokyo (Tokyo, Japan; approval number 402-2), and the Riken National Science Institute (Wakō, Saitama Prefecture, Japan; approval number 16-27) and performed in accordance with the guidelines and regulations of the institute(s). All participants provided written informed consent before participation, and participant anonymity was preserved.

### 2.2. Psychological Scales

The Trait Curiosity and Exploration Inventory II scale developed by Kashdan et al. [14] contains 10 items, including “I actively seek as much information as I can in new situations”. The Short Grit Scale, originally created by Duckworth et al. [41] and afterward by Duckworth and Quinn [15], contains eight items, including “I finish whatever I begin”. Self-efficacy was measured using the General Self-Efficacy Scale (GSE) created by Sherer et al. [29], which contains 23 items, including “When I make plans, I am certain I can make them work”. Self-esteem was measured using the Rosenberg Self-Esteem Scale developed by Rosenberg et al. [30], which contains 10 items, including “On the whole I am satisfied with my-self”. Optimism was assessed using the 10-item Life Orientation Test developed by Scheier et al. [31], which contains six items (four of which were “filler”), including “In uncertain times, I usually expect the best”. Participants responded to these items on a 5-point Likert scale, except for those pertaining to self-esteem, which were answered on a 4-point scale. The consistencies of four of the five variables scored >0.7, which is a generally acceptable level [42]. Although the consistency of optimism was <0.7, it increased from 0.688 to 0.722 by excluding one of six composing items: “If something can go wrong for me, it will”. Therefore, another complementary composition (excluding this item) was used for this variable in the following analysis, although it is not reported in the tables. The summary of these questionnaires is shown in Table 1. The scale scores were calculated by averaging answered figures to response scales.

### 2.3. MRI Data Acquisition

All MRI data were collected using a 3 Tesla Siemens scanner (Verio, Siemens Medical Solutions, Erlangen, Germany or MAGNETOM Prisma, Siemens, Munich, Germany) equipped with a 32- or 64-channel head array coil at Riken, Kyoto University, and the University of Tokyo. A high-resolution structural image was acquired using a 3D T1-weighted magnetization-prepared rapid-acquisition gradient echo (MP-RAGE) pulse sequence. The parameters were as follows: repetition time (TR), 1900 ms; echo time (TE), 2.52 ms; inversion time (TI), 900 ms; flip angle, 9°; matrix size, 256 × 256; field of view (FOV), 256 mm; slice thickness, 1 mm.

### 2.4. MRI Data Analysis

The calculation of the GM-BHQ was similar to the method described by Nemoto et al. [12]. Briefly, gray matter images were segmented from T1-weighted images using Statistical Parametric Mapping 12 (SPM12; Wellcome Trust Centre for Neuroimaging, London, UK) running on MATLAB R2015b (Mathworks Inc., Sherborn, MA, USA), followed by spatial normalization using diffeomorphic anatomical registration through an exponentiated lie algebra (DARTEL) algorithm [43] and modulation to preserve GMV. All normalized, segmented, and modulated images were smoothed using an 8 mm full-width at half-maximum (FWHM) Gaussian kernel. Additionally, intracranial volume (ICV) was calculated by summing the gray matter (GM), white matter, and cerebrospinal fluid images for each subject. Proportional GM images were generated by dividing smoothed GM images by ICV to control for differences in whole-brain volume across participants. Using these proportional GM images, images for the mean and SD across participants were generated. The GM-BHQ was then calculated using the following formula: 100 + 15 × (individual proportional GM – mean) / SD. Regional GM quotients were then extracted using the automated anatomical labeling (AAL) atlas [11] and averaged across regions to produce participant-specific GM-BHQ.

### 2.5. Statistical Analysis

Correlation analysis was used to investigate the association between the GM-BHQ and various variables based on the hypothesis that ambidexterity scale variables (i.e., curiosity and grit) are related to GM-BHQ. The level of statistical significance was set at *p* < 0.05. All statistical analyses were performed using SPSS version 26 (IBM Corporation, Armonk, NY, USA).

## 3. Results

Table 2 indicates that there was statistical mean difference between men and women for GM-BHQ (*t* = 4.872, *p* < 0.001) according to the Student’s *t* test. There was also statistical distributional difference between men and women for the places of participation (χ^2^ = 9.844, *p* < 0.01) according to the results of the chi-squared test. Likewise, Table 3 indicates that there were statistical differences among three places for self-esteem (F (2, 197) = 3.726, *p* < 0.05) and age (F (2, 197) = 4.364, *p* = 0.05) according to the analysis of variance (ANOVA). However, there was no statistical mean difference in hypothesized scales (GM-BHQ, curiosity, and grit) among places. Therefore, we reached a decision to use the entire sample in a single model controlling for age and sex in the following analyses. For reference, analyses controlled for places of participation (with age and sex) were also conducted but did not alter the results significantly (available upon request).

Descriptive statistics of all subjects and correlation coefficients between the psychological scales are shown in Table 4. GM-BHQ was correlated only with age (*r* = −0.763, *p* < 0.001), sex (*r* = 0.324, *p* < 0.001), and self-esteem (*r* = −0.140, *p* < 0.05) as appeared below diagonal. However, GM-BHQ was correlated with curiosity (*r* = 0.184, *p* < 0.01), grit (*r* = 0.151, *p* < 0.05), and self-efficacy (*r* = 0.155, *p* < 0.05) but not with self-esteem (*r* = −0.002, *p* > 0.05) and optimism (*r* = 0.048, *p* > 0.05) after controlling for age and sex as appeared above diagonal. For reference, the result was not significantly different for the abovementioned other version of optimism (*r* = 0.058. *p* = 0.416) after the control. Partial correlation coefficients of three psychological scales (curiosity, grit, and self-efficacy) were higher than 0.10, an effect size that is “small”, but lower than 0.30, an effect size that is “moderate”, by Cohen’s criterion [44]. Therefore, it is safe to say that ambidexterity scales are associated with GM-BHQ even though the magnitude is relatively small.

**Figure 2:**
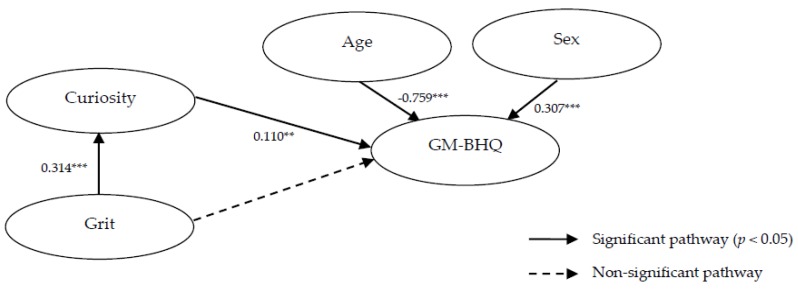
Path diagram for the resulting association between psychological scales and the GM-BHQ. Goodness-of-fit indices: χ^2^ = 5.874; *df* = 5; root mean square error of approximation (RMSEA) = 0.030; probability of close fit (PCLOSE) = 0.576; goodness of fit index (GFI) = 0.989; adjusted goodness of fit index (AGFI) = 0.966; normed fit index (NFI) = 0.978; comparative fit index (CFI) = 0.997. *n* = 200; ** *p* < 0.01; *** *p* < 0.001.

To compare the strengths and priorities in the effect of GM-BHQ between ambidexterity variables, we also conducted path analysis, as depicted in Figure 2. For reference, the figures of the standardized path coefficient, calculated using AMOS Version 26 (IBM Corp., Armonk, NY, USA), are also shown. The model’s goodness-of-fit indices (displayed under the figure) showed high adaptability. However, although curiosity had a direct association with GM-BHQ, grit did not; grit had only an indirect association with GM-BHQ via curiosity. Therefore, the total effect on GM-BHQ was 0.110 for curiosity, while it was 0.035 for grit, as shown in Table 5. For reference, the results were not so altered when we used self-efficacy instead of grit in the path model (available upon request). The results are summarized as follows: First, curiosity, grit, and self-efficacy demonstrated positive partial correlations with GM-BHQ after adjusting for age and sex, even though their effect sizes were relatively small. Second, among these, curiosity demonstrated the highest and a direct positive association with GM-BHQ. Third, grit had an indirect and positive association with GM-BHQ via curiosity.

## 4. Discussion

Ambidexterity is a dynamic capability that enables an individual or organization to switch between explorative and exploitative behaviors, which can lead to innovation and appropriate decision-making. Exploration requires detachment from present duties in order to experiment freely, allowing for new discoveries and innovations, while exploitation is about focusing on the current endeavor in order to improve or maximize benefits or opportunities [10]. In fact, a mounting number of studies indicate that ambidexterity is associated not only with work performance but also with the human brain [7,8,9,10]. For example, using a gambling task, Daw et al. [9] reported that the frontopolar cortex and intraparietal sulcus were activated during exploratory or speculative behaviors; in contrast, areas of the striatum and ventromedial prefrontal cortex exhibited activation during exploitative decision-making. In the same vein, using a task in which subjects can either speculate (i.e., exploration) or take risks (i.e., exploitation), Blanchard and Gershman [8] found that the insula and dorsal anterior cingulate cortex exhibited significantly greater activation among speculative trials compared with trials that were risk-taking, suggesting that these areas of the brain support exploratory behavior.

Up until now, however, there have not been any definitive MRI-based parameters than can objectively and easily evaluate ambidexterity levels. In this research, we used the GM-BHQ―an MRI-based quotient for monitoring brain health based on GMV [12]―as an objective measure to evaluate the association of ambidexterity with the entire brain. Through the analysis of the relationships between a healthy participant’s GM-BHQ and the results of exploration (i.e., curiosity) and exploitation (i.e., grit) scales, we found that GM-BHQ seemed to be high in individuals with high scores on these scales after controlling for age and sex, even though these effects were relatively small. Moreover, we could find the differences in priorities between the scales. Curiosity had a direct and stronger association with GM-BHQ than grit, which was only indirectly associated with GM-BHQ. Therefore, the total effect of curiosity was almost threefold higher than grit. In other words, these results indicate a weak but significant association between gray matter of the entire brain and high ambidexterity, with a stronger association of exploration (curiosity) than exploitation (grit).

Previous research has suggested that curiosity may stimulate and sustain not only work-related behavior, such as job performance [19] and worker innovation [20], but also subjective well-being [45]. In support, in the field of neuroscience, it has been demonstrated that curiosity is associated with activity in the hippocampus, brain circuit, the lateral prefrontal cortex, and the caudate, which are recognized as areas partly responsible for creating memories or related to reward and pleasure [46,47]. Moreover, other research has demonstrated an association between curiosity and gray matter density in the precuneus [21] or frontal GMV [48]. Grit has also been found to be positively associated with successful completion of a training course, job, or study continuity [22]; academic and/or career success [22,23,24,25]; and overall life satisfaction and happiness [49], and negatively associated with anxiety [50] and depression [51]. In the field of neuroscience, it has been suggested that grit leads to higher academic performance through the neural link of the right dorsomedial prefrontal cortex, a highly associative center in the frontal cortex [23,52]. In other research, high grit was associated with greater regional GMV in the right putamen, an area known to be involved in reward-based motivation and learning [27,28].

By extension, another experimental study reported a link between grit and exploration, demonstrating that individuals higher in grit were more likely to persist with an impossible task [17]. Similarly, other research has indicated positive effects of self-efficacy on both exploration and exploitation [53]. These multiassociations are important because sustained high levels of performance depend on an individual’s ability to shift between exploratory and exploitative behavior, which at the same time is influenced by strong activity in certain regions of the brain (specifically those responsible for attention and cognitive control) [10]. The brain regions most often associated with the explore–exploit dilemma are the dorsolateral and medial prefrontal cortices, which also interface with different brain regions associated with selective attention, action evaluation, and outcome prediction. This includes the anterior cingulate cortex; the hippocampal formation; and the dorsal, ventral, medial, and lateral aspects of the striatum, governed by numerous neuromodulators such as dopamine, acetylcholine, and noradrenaline [54].

The results of this study are therefore consistent with the outcomes of preceding studies, and at the same time, offer new insights, such as that the condition of the entire brain measured by the GM-BHQ is weakly but positively related to curiosity and grit. These findings are meaningful because they indicate that individuals with good whole-brain health, according to the GM-BHQ, tend to have high ambidexterity, including explorative and exploitative abilities, even though the effect may be relatively small. Although previous research has identified noteworthy implications of an organization’s learning environment on an individual’s explore–exploit behaviors and creativity [55], the results of our research indicate the possibility of adding a biological approach to this process. For example, our previous research suggested a link between fatigue/stress and brain health [13]. Similarly, our other research has indicated an association between dietary intake and brain health [56]. Therefore, we may develop wider protocols to focus not only on learning but also on welfare, such as arrangement of recess, enrichment of health control, and enhancement of nutrition, to increase individual creativity, which can be an important competitive advantage [57]. Moreover, our results may contribute to advances in research investigating artificial intelligence because the evolutionary algorithm, a component of evolutionary computation, may be enhanced by a deeper understanding of the explore–exploit dilemma, which may shed light on why behaving a certain way (e.g., switching to an exploratory behavior) in a particular setting is better than staying in an exploitative behavior [58].

Additionally, our results demonstrated a positive association between GM-BHQ and self-efficacy (i.e., confidence in one’s ability to achieve a desired outcome) [32], contradicting our hypothesis depicted in Figure 1. However, previous experimental research has indicated positive effects of self-efficacy on both exploration and exploitation [53], which is similar to the association between grit and exploration found by Dale et al. [17]. In support, self-efficacy was associated with better performance in studies [33], sport [34], work [35,59], and health-promoting behaviors involving dietary habits and exercise [36], medication adherence [60], and patients’ mental and physical health [61]. Self-efficacy has been reported to be significantly and positively correlated with GMV in the left posterior insular cortex [62] and the posterior precuneus [63]. Therefore, self-efficacy may have similar or complementary characteristics to grit and, therefore, demonstrate associations with the brain and behaviors.

GM-BHQ, however, was not associated with self-esteem and optimism in the current research. One possible explanation is that these measures are used to assess positive *feelings* only and may be weak in the link to exploration and exploitation, being different from other variables. Another possible explanation is that the GM-BHQ, a scale for measuring the health of the *entire* brain, is not sensitive to *regional* conditions. Therefore, although the relationship between these psychological scales and several brain regions have been elucidated in previous research [64], such associations at the level of the entire brain may be weaker than those observed for variables of ambidexterity.

There were two particular limitations to the present study. First, observing the association of brain health with actual activities, attitudes, and performances may have improved the validity of our findings. Second, a larger number of samples may have increased the generalizability of the findings. Nevertheless, prospective investigations exploring the link between GM-BHQ and actual behaviors using larger sample sizes are warranted in order to further elucidate the mechanisms connecting these two variables.

## Figures and Tables

**Table 1:** Description of the scales used in this research.

Scale	Number of Items Comprising the Scale	Cronbach’s α	Original Name	Response Scale	Sample Item	Source
Curiosity	10	0.898	Trait Curiosity and Exploration Inventory II	5 points from 1 (very slightly or not at all) to 5 (extremely)	I actively seek as much information as I can in new situations.	Kashdan et al. [14]
Grit	8	0.777	Short Grit Scale	5 points from 1 (not like me at all) to 5 (very much like me)	Setbacks don’t discourage me.	Duckworth and Quinn [15]
Self-efficacy	23	0.906	General Self-Efficacy Scale	5 points from 1 (strongly disagree) to 5 (strongly agree)	When I make plans, I am certain I can make them work.	Sherer et al. [29]
Self-esteem	10	0.873	Rosenberg Self-Esteem Scale	4 points from 1 (strongly disagree) to 4 (strongly agree)	On the whole, I am satisfied with myself.	Rosenberg et al. [30]
Optimism	6	0.688	Life Orientation Test	5 points from 1 (strongly disagree) to 5 (strongly agree)	In uncertain times, I usually expect the best.	Scheier et al. [31]

**Table 2:** Statistical differences between male and female participants.

	Male		Female			
	Mean	SD	Mean	SD	*t*	*p*
GM-BHQ	98.003	8.868	103.660	7.613	4.827	***
Curiosity	2.610	0.791	2.487	0.740	1.135	
Grit	3.267	0.660	3.273	0.540	0.072	
Self-efficacy	3.314	0.622	3.360	0.501	0.567	
Self-esteem	2.889	0.563	2.900	0.525	0.139	
Optimism	3.175	0.601	3.270	0.548	1.166	
Age	44.864	13.462	43.866	10.775	0.577	
	*n*	%	*n*	%	*χ2*	
Kyoto	57	55.3	58	59.8	9.844	**
Tokyo	23	22.3	32	33.0		
Kobe	23	22.3	7	7.2		

*n* = 200; ** *p* < 0.01; *** *p* < 0.001.

**Table 3:** Statistical differences among places for participation.

	Kyoto		Tokyo		Kobe			
	Mean	SD	Mean	SD	Mean	SD	*F* (2, 197)	*p*
GM-BHQ	101.199	9.936	99.213	6.047	101.828	7.806	1.235	
Curiosity	2.528	0.779	2.484	0.709	2.757	0.811	1.348	
Grit	3.266	0.640	3.227	0.525	3.363	0.603	0.490	
Self-efficacy	3.356	0.554	3.282	0.624	3.362	0.505	0.349	
Self-esteem	2.929	0.530	2.740	0.568	3.047	0.497	3.726	*
Optimism	3.248	0.607	3.091	0.566	3.356	0.426	2.375	
Age	43.348	14.180	48.273	6.066	41.200	11.006	4.364	*

*n* = 200; * *p* < 0.05.

**Table 4:** Descriptive statistics and correlations.

	Variable	Mean	SD	1	2	3	4	5	6
1	GM-BHQ	100.747	8.735		0.188 **	0.154 *	0.155 *	0.002	0.052
2	Curiosity	2.550	0.767	0.105		0.328 ***	0.555 ***	0.333 ***	0.277 ***
3	Grit	3.270	0.604	-0.060	0.314 ***		0.662 ***	0.469 ***	0.215 **
4	Self-efficacy	3.337	0.566	-0.062	0.529 ***	0.676 ***		0.674 ***	0.459 ***
5	Self-esteem	2.895	0.543	-0.140 *	0.319 ***	0.490 ***	0.687 ***		0.569 ***
6	Optimism	3.221	0.576	0.047	0.268 ***	0.213 **	0.453 ***	0.560 ***	
7	Age	44.380	12.213	-0.763 ***	-0.028	0.197 **	0.214 ***	0.192 **	0.009

*n* = 200; * *p* < 0.05; ** *p* < 0.01; *** *p* < 0.001; correlations appear below diagonal and partial correlations (controlled for age and sex) above diagonal.

**Table 5:** Path coefficient and direct/indirect effect.

			Effect (standardized)		
Path			Direct	Indirect	Total
Curiosity	→	GM-BHQ	0.110		0.110
Grit	→	Curiosity	0.314		0.314
Age	→	GM-BHQ	−0.759		−0.759
Sex	→	GM-BHQ	0.307		0.307
Grit	→	GM-BHQ		0.035	0.035
